# Targeting SARS-CoV-2 main protease for the discovery of a broad-spectrum COVID-19 inhibitor by intensive multi-tiered validation

**DOI:** 10.1016/j.apsb.2025.09.033

**Published:** 2025-09-22

**Authors:** Min Zhang, Changjian Wang, Lu Feng, Qi Yang, Yipeng Cao, Yao Zhao, Junhua Zhang, Yuefei Wang, Zihe Rao, Boli Zhang

**Affiliations:** a State Key Laboratory of Chinese Medicine Modernization, Tianjin University of Traditional Chinese Medicine, Tianjin 301617, China; b Haihe Laboratory of Modern Chinese Medicine, Tianjin 301617, China; c State Key Laboratory of Medicinal Chemical Biology, Frontiers Science Center for Cell Response, College of Life Sciences, College of Pharmacy, Nankai University, Tianjin 300071, China; d Guangzhou Laboratory, Guangzhou 510005, China; e Key Laboratory of Cancer Prevention and Therapy, Tianjin's Clinical Research Center for Cancer, National Clinical Research Center for Cancer, Tianjin Medical University Cancer Institute and Hospital, Tianjin 300060, China; f National Clinical Research Center for Infectious Disease, Shenzhen Third People's Hospital, Shenzhen 518112, China; g Shanghai Institute for Advanced Immunochemical Studies and School of Life Science and Technology, ShanghaiTech University, Shanghai 200031, China; h Laboratory of Structural Biology, School of Life Sciences and School of Medicine, Tsinghua University, Beijing 100084, China

**Keywords:** COVID-19, SARS-CoV-2, Main protease (M^pro^), Broad-spectrum antiviral activity, Chebulagic acid (CHLA), Allosteric inhibitor, X-ray crystallography, Molecular interaction

## Abstract

SARS-CoV-2 and its emerging variants continue to pose a significant global public health threat. The SARS-CoV-2 main protease (M^pro^) is a critical target for the development of antiviral agents that can inhibit viral replication and transcription. In this study, we identified chebulagic acid (CHLA), isolated from *Terminalia chebula* Retz., as a potent non-peptidomimetic and non-covalent M^pro^ inhibitor. CHLA exhibited intermolecular interactions and provided significant protection to Vero E6 cells against a range of SARS-CoV-2 variants, including the wild-type, Delta, Omicron BA.1.1, BA.2.3, BA.4, and BA.5, with EC_50_ values below 2 μmol/L. Moreover, *in vivo* studies confirmed the antiviral efficacy of CHLA in K18-hACE2 mice. Notably, CHLA bound to a unique groove at the interface between M^pro^ domains I and II, which was revealed by the high-resolution crystal structure (1.4 Å) of the M^pro^–CHLA complex, shrinking the substrate binding pocket of M^pro^ and inducing M^pro^ aggregation. CHLA was proposed to act as an allosteric inhibitor. Pharmacokinetic profiling and safety assessments underscore CHLA's potential as a promising broad-spectrum antiviral candidate. These findings report a novel binding site on M^pro^ and identify antiviral activity of CHLA, providing a robust framework for lead compounds discovery and elucidating the underlying molecular mechanisms of inhibition.

## Graphical abstract

This study reported a novel binding site on M^pro^ and identified the antiviral activity of chebulagic acid, providing a robust framework for lead compounds discovery and elucidating the underlying molecular mechanisms of inhibition.

**Figure:**
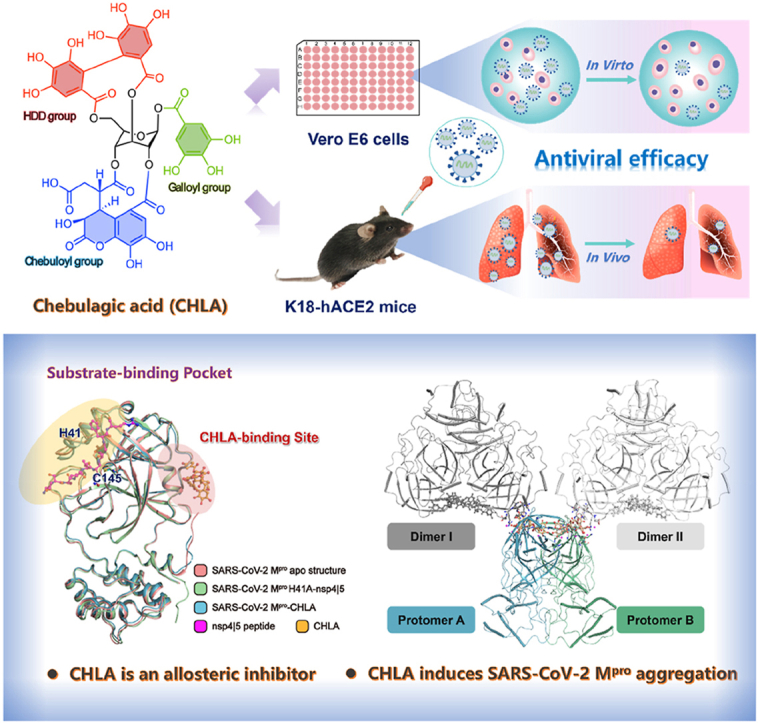

## 1 Introduction

The ongoing prevalence of COVID-19 caused by severe acute respiratory syndrome coronavirus 2 (SARS-CoV-2) and variants has highlighted the immediacy of the development of specific antiviral drugs. So far, more than 774 million cases have been confirmed, with more than 7.04 million deaths reported by the World Health Organization (WHO). Within the past three years, some antiviral drugs have been approved for the treatment of COVID-19, including Remdesivir and Molnupiravir as inhibitors targeting RNA-dependent RNA polymerase (RdRp)^1^^,^^2^, Paxlovid and Xocova (Ensitrelvir) as inhibitors targeting the SARS-CoV-2 main protease (M^pro^)^3^, and Tocilizumab targeting interleukin-6 receptor (IL-6)^4^. However, some of them cannot satisfy the clinical practice due to the high risk of safety and drug resistance^5^, ^6^, ^7^. In order to conquer resurgent SARS-CoV-2 in the future, it is urgent to discover and reserve more effective and biosafe broad-spectrum antiviral drugs^8^, ^9^, ^10^, ^11^, ^12^.

As a typical proteolytic enzyme, SARS-CoV-2 M^pro^ is focused on its key function for virus replication by cleaving the polyprotein into functional units. By taking high conservation and the absence of homologous protein in the host cells into account, M^pro^ becomes a crucial druggable target for the discovery of a broad-spectrum anti-coronavirus drug, which belongs to the C30 family of proteolytic enzymes^13^ and displays similarity to the sequence of SARS-CoV M^pro^. Three distinct domains were identified, including domain I (residues 8–101), domain II (residues 102–184), and domain III (residues 201–303). The crucial catalytic dyad (H41 and C145) is located at the bottom of the substrate binding pocket formed in the cleft between domain I and domain II. The formed cavities are the essential binding sites for the specific ligands of M^pro^^14^. Chinese Materia Medica (CMM) is a natural treasure for treating numerous diseases, especially infectious diseases. The diversified compounds from CMM are critical resources for antiviral drug discovery and development^15^, ^16^, ^17^, benefiting from their skeleton diversity and structural complexity.

In our study, chebulagic acid (CHLA), as a phenolic acid purified from *Terminalia chebula* Retz. in our lab^18^, was fortunately identified as a candidate against SARS-CoV-2. The fluorescence resonance energy transfer (FRET) assay was employed to confirm the inhibitory activity on SARS-CoV-2 M^pro^. The thermal shift assay (TSA), surface plasmon resonance (SPR), and spectral shift (SpS) analysis were undertaken to provide insights into the robust binding interactions between SARS-CoV-2 M^pro^ and CHLA. Importantly, by the cellular assays, the approved antivirus activity was demonstrated against SARS-CoV-2 wild-type strain (WT), Delta strain, and Omicron variants (BA.1.1, BA.2.3, BA.4, and BA.5). Additionally, *in vivo* studies confirmed the antiviral efficacy of CHLA in K18-hACE2 mice. Satisfactorily, the crystal structure of SARS-CoV-2 M^pro^–CHLA complex with a high resolution of X-ray diffraction was obtained at 1.4 Å. It is clearly superior to the reported natural SARS-CoV-2 M^pro^ inhibitors, shikonin^19^ and baicalein^20^, with the resolution of X-ray diffraction at 2.25 and 2.28 Å. CHLA binds to a unique groove at the interface between M^pro^ domains I and II, which differs from the traditional substrate-binding pocket, shrinking the substrate-binding pocket of SARS-CoV-2 M^pro^. Moreover, CHLA may function in a lectin-like manner, promoting M^pro^ aggregation and thereby impairing its function to inhibit viral replication as an allosteric inhibitor. Importantly, these findings suggested a novel binding pocket and binding mode, which are essential for inhibitor screening and the disclosure of the antiviral mechanism. Moreover, the pharmacokinetic properties and safety assessments *in vivo* were implemented to study druggability. Altogether, this study not only identifies a novel broad-spectrum SARS-CoV-2 M^pro^ inhibitor but also provides a robust framework for lead compound discovery and elucidates the underlying molecular mechanisms of inhibition.

## 2 Materials and methods

### 2.1 Chemicals and reagents

CHLA and PF-07321332 were acquired from Shanghai Yuanye Bio-Technology Co., Ltd. (Shanghai, China). All procedures involving animal care and experimentation were conducted in strict adherence to the Guide for the Care and Use of Laboratory Animals (National Institutes of Health). These protocols received approval from the Animal Care and Use Committee of Tianjin University of Traditional Chinese Medicine (Approval Nos: TCM-LAEC2022003 and TCM-LAEC2023135).

### 2.2 Construction of plasmid, expression, and purification of SARS-CoV-2 M^pro^

The plasmid, which encodes the full-length gene of SARS-CoV-2 M^pro^ (NC_045512), was transferred into *Escherichia coli* BL21 (DE3) cells and cultured in Luria broth medium with 100 μg/mL ampicillin at 37 °C, 220 rpm for 6–8 h (ZhiChu Orbital Shaking Incubator, Shanghai, China). When the OD_600_ reached 0.6–0.8, 500 μmol/L IPTG was added to the cell culture flasks to trigger the expression of M^pro^ for 16–20 h at 16 °C. The cells were harvested through centrifugation at 3500 rpm (Beckman Coulter avanti J-25, USA), and the pellets were resuspended in running buffer (20 mmol/L Tris-HCl, pH 8.0, 150 mmol/L NaCl, and 5% Glycerol). Then, the cells were lysed by a high-pressure homogenizer at 4 °C and centrifuged at 18,000 rpm for 30 min (Beckman Coulter avanti J-25, USA). Subsequently, the supernatant containing the recombinant protein was purified *via* a Ni-NTA affinity chromatography column (Qiagen, Germany). The running buffer with various concentrations of imidazole eliminates impurities and isolates the desired protein. A 6 × His tag, engineered at the C-terminus of the protein, can be cleaved by the human rhinovirus 3C protease. Finally, purification was performed using a gel filtration column (Superdex 200 Increase 10/300 GL, GE Healthcare Life Sciences, Cytiva, USA), ensuring the attainment of high-purity SARS-CoV-2 M^pro^ suitable for further studies. Purified M^pro^ was concentrated to 14 mg/mL and stored at −80 °C.

### 2.3 Enzyme inhibition activity

The substrate of SARS-CoV-2 M^pro^ is specifically designed and synthesized as MCA-AVLQSGFR-Lys (Dnp)-Lys-NH_2_ by GL Biochem Co., Ltd. (Shanghai, China), which is adept at selectively identifying proteins by modifying the proximity between the fluorescent receptor and the donor. In order to determine the IC_50_ value of CHLA, a high-throughput screening approach tailored for enzyme inhibitor identification was established. SARS-CoV-2 M^pro^ was solved in a buffer (20 mmol/L Tris-HCl, pH 8.0, 4 mmol/L DTT, and 5% glycerol), and introduced into each well at a final concentration of 200 nmol/L. Then, CHLA was serially diluted in the same buffer to final concentrations ranging from 0.004 to 320 μmol/L. After incubating for 1 h at room temperature, 5 μL of the substrate (20 μmol/L) was added to interact with unbound M^pro^^21^. The fluorescence was quantified using an excitation wavelength of 320 nm and an emission wavelength of 405 nm. PF-07321332, identified as the SARS-CoV-2 M^pro^ inhibitor^22^ in prior studies, served as the positive control. Data analysis was conducted with GraphPad Prism 8.0 software, and all experiments were performed in triplicate.

### 2.4 TSA assay

SARS-CoV-2 M^pro^ was prepared in a buffer (20 mmol/L Tris-HCl, pH 8.0, and 150 mmol/L NaCl), and CHLA was dissolved in DMSO. These were mixed to yield final concentrations of 10 μmol/L for M^pro^ and 500 μmol/L for CHLA, incubating for 15 min at 25 °C. Subsequently, it was manually loaded into standard nano-differential scanning fluorimetry (nano DSF) grade capillaries (NanoTemper Technologies). The thermal stability of the protein was assessed using a Prometheus nano-DSF instrument, employing a temperature gradient from 20 to 95 °C at a rate of 1 °C/min. The unfolding process of the protein was monitored by analyzing the shift in the rate of fluorescence emission wavelengths at 350 and 330 nm, indicating the changes in the environmental conditions surrounding tryptophan and tyrosine residues.

### 2.5 SPR assay

SPR assay was explored to investigate the interaction between the ligands and a protein by the Biacore T200 system (GE Healthcare). The protein, acting as the receptor, was immobilized on a CM5 sensor chip surface *via* EDC/NHS-mediated amino coupling reaction, and the molecules, as the ligands, flowing over the sensor chip surface continuously, would bind to the receptor. When the ligands bound to the receptor, they would induce changes in the local reflective index and alter the resonance conditions of the surface plasmon waves. The compound was diluted in a working buffer (PBS-P, 5% DMSO) at concentrations ranging from 0.24 to 15.6 μmol/L and injected into channels at a flow rate of 10 μL/min with a temperature of 25 °C. The association and dissociation phases were timed at 120 and 180 s, respectively. The data were analyzed by Biacore T200 evaluation software with a steady state affinity model to obtain the equilibrium *K*_D_.

### 2.6 SpS measurements

SARS-CoV-2 M^pro^ was labeled utilizing the Monolith His-Tag Labeling Kit RED-tris-NTA 2nd Generation (NanoTemper Technologies) following the manufacturer's guidelines. For the binding assays, CHLA served as a ligand across three parallel two-fold dilution series, initiating at concentrations of 20 μmol/L specifically for labeled SARS-CoV-2 M^pro^ targets. The concentrations of the targets were maintained at a steady 100 nmol/L for each dilution. Ratiometric measurements were carried out on a Monolith X instrument (NanoTemper Technologies) at wavelengths 670 and 650 nm, within 10 min window post-binding assay preparation. The ratiometric approach of the spectral-shift technology allows focusing the measurement on the active fraction of the target, thereby enhancing the signal-to-noise ratio for samples that present a challenge^23^. The *K*_D_ was determined on OriginPro (2022, OriginLab Corporation), employing curve fitting on the acquired data by Hill's equation, with Hill coefficients set to 1.

### 2.7 Analysis of NMR spectra

NMR spectra were employed to demonstrate the hydrogen bonding between the CHLA and SARS-CoV-2 M^pro^, as evidenced by both STD and Water-LOGSY. CHLA and M^pro^ were individually dissolved in D_2_O to prepare the sample solutions for STD (*C*_CHLA_:*C*_Mpro_ at about 100:1) and WaterLOGSY (*C*_CHLA_:*C*_Mpro_ at about 10:1) analysis. After centrifuging for 5 min at 6000 rpm (Thermo Scientific Sorvall ST 40R Centrifuge, Osterode, Germany), the supernatant was transferred to 5 mm NMR tubes for testing, using a 600 MHz NMR spectrometer (Norrel, Inc., USA). The acquisition parameters were set as follows: the number of scans was 256, the temperature was 298 K, the spectral width was 16 ppm, and the recycle delay time ranged from 1.5 to 10 s. The data were analyzed by Mestrenova software (Version 9.0).

### 2.8 Cell viability assay

Cell viability was assessed using the Cell Counting Kit-8 assay (CCK-8, MedChemExpress, China). In brief, Vero E6 cells (ATCC, CRL-1586) at a density of 5 × 10^4^ cells/well were seeded into 96-well plates for 24 h. Cells were cultured in Dulbecco's modified Eagle's medium (DMEM) (Gibco, USA), supplemented with 10% (*v*/*v*) serum, 100 U/mL penicillin, and 100 μg/mL streptomycin. Serial dilutions of CHLA (200 μL) at concentrations of 100, 80, 60, 40, 20, 10, and 5 μmol/L were added to wells and incubated for another 24 or 48 h, respectively. Following the incubation, the existing medium was replaced with fresh medium containing a 10% CCK-8 solution and incubated at 37 °C for 1 h. Absorbance at 450 nm was measured using a Microplate Reader (TECAN Sunrise™) to determine the final optical density. Data were analyzed using GraphPad Prism 8.0 software (version 8.0). All experiments were performed in triplicate to ensure reliability.

### 2.9 Antiviral assay *in vitro* and *in vivo*

The antiviral infection studies were conducted at an Association for Assessment and Accreditation of Laboratory Animal Care (AAALAC) accredited animal biosafety level 3 (ABSL3) laboratory at Guangzhou Customs Inspection and Quarantine Technology Center (IQTC), ensuring compliance with the highest safety and operational standards.

*In vitro*, Vero E6 cells (ATCC, CRL-1586) were seeded in 96-well plates at a density of 2 × 10^4^ cells per well within a fully humidified cultivation chamber at 37 °C containing 5% CO_2_ and under bacteria-free conditions. These cells were exposed to various SARS-CoV-2 variants at a multiplicity of infection (MOI) of 0.01, including the WT, Delta, Omicron BA.1.1, Omicron BA.2.3, Omicron BA.4, and Omicron BA.5, along with varying concentrations of CHLA (ranging from 0.0137 to 30.00 μmol/L). After 48 h incubation, a cytopathic effect (CPE) was assessed using the Celigo Image Cytometer. The degree of CPE was quantitatively scored to determine the half-maximal effective concentration (EC_50_) values, thereby determining the antiviral activity of the tested compounds.

*In vivo*, a total of 20 female K18-hACE2 transgenic mice aged 8 weeks were obtained from GemPharmatech (Nanjing, China), which were classified into four groups with five mice in each group: Group 1 served as the untreated, infected control (Virus group); Group 2 received 300 mg/kg of PF-07321332 as the positive control; and Groups 3 and 4 were administered CHLA at doses of 150 and 300 mg/kg, respectively. On Day 0, these K18-hACE2 mice were intranasally inoculated with 1000 FFU of SARS-CoV-2 WT, which was pre-diluted in 50 μL of DMEM. The therapeutic administration was commenced 2 h after infection. The K18-hACE2 transgenic mice were orally administered with either PF-07321332 (300 mg/kg) or CHLA at varying doses (150 and 300 mg/kg), which were dissolved in a saline solution containing 5% DMSO. The mice were euthanized three days post-administration, and the lungs were collected and placed in 1 mL PBS and preserved at −80 °C to evaluate pulmonary virus titers.

### 2.10 Crystallization, data collection, and structure determination

Purified SARS-CoV-2 M^pro^ was diluted to a concentration of 6 mg/mL in a buffer of 20 mmol/L Tris-HCl, pH 8.0. The protein was incubated with 1 mmol/L CHLA for 1 h at room temperature, then the complex was crystallized at 16 °C by using the hanging-drop vapor-diffusion method. Droplets were formed from 1 μL of protein and a 1 μL reservoir, which were equilibrated with 300 μL of the solution. The collected crystals were grown in reservoir solutions containing 0.1 mol/L MES pH 5.4, 11% (*w*/*v*) PEG 6000, and 6% DMSO. The cryo-protectant solution is formed by 80% reservoir and 20% glycerol.

X-ray diffraction data were collected on beamline BL10U2 at Shanghai Synchrotron Radiation Facility (SSRF) at 100 K with a wavelength of 0.97918 Å using an Eiger X 16M image plate detector. Data integration and scaling were performed using the XDS software^24^. The structure was determined by molecular replacement (MR) using PHASER^25^ and Phenix 1.21.1 for analysis^26^. The structure of SARS-CoV-2 M^pro^ in complex with N3 (PDB ID: 6LU7) was used as a search template. Subsequently, the model building and refinement were iteratively conducted with Coot (version 0.8.9.2)^27^ and Phenix.refine^28^, respectively. The inhibitor CHLA was modeled based on the corresponding omit map. Detailed phasing and refinement metrics are provided in Supporting Information Table S1. Coordinates and structure have been deposited in the PDB.

### 2.11 Pharmacokinetic (PK) study

The PK study was strictly conducted according to the National Medical Products Administration (NMPA) Good Laboratory Practice (GLP) guidelines, which was undertaken at the Animal Center of Tianjin University of Traditional Chinese Medicine. Sprague–Dawley (SD) rats were supplied by Beijing Vital River Laboratory Animal Technology Co., Ltd. (Beijing, China). Male SD rats (*n* = 5 per group, weights: 180–200 g and age: 6–8 weeks) were administered by CHLA (solubilized in DMSO: 0.9% NaCl = 5:95, *v*/*v*) at doses of 10 and 5 mg/kg *via* intragastric (i.g.) and intravenous (i.v.) routes, respectively. Blood samples were collected at specified intervals post-administration (0.033, 0.083, 0.166, 0.25, 0.5, 1, 4, 6, 8, 12, 24, 36, and 48 h), followed by centrifugation at 4000 rpm (Thermo Scientific Sorvall ST 40R Centrifuge, Osterode, Germany) and 4 °C for 10 min. The supernatant plasma was stored at −20 °C for subsequent analysis.

Analysis was performed on a Shimadzu LC-20AD Prominence Liquid Chromatograph (Shimadzu Corporation, Japan)–AB Sciex API 4000→Qtrap triple quadrupole bar mass spectrometer (Applied Biosystems, USA) (LC–MS/MS). Samples were separated on an Agilent Poroshell 120 EC-C18 column (2.7 μm, 3.0 mm × 50 mm) (Agilent, USA). The column temperature was 35 °C, and the mobile phase consisted of water with 0.1% formic acid (*v*/*v*, buffer A) and acetonitrile with 0.1% formic acid (*v*/*v*, buffer B). The elution was conducted according to a 5-min gradient program (0–0.5 min, 10% buffer B; 0.5–2 min, 10%–90% buffer B; 2–3.5 min, 90% buffer B; 3.5–5 min, 10% buffer B), with the flow rate set at 0.5 mL/min and the injection volume at 10 μL. The mass spectrometry operated in positive MRM mode, targeting the precursor and product ions of CHLA (*m/z* 953.3 →301.1 and 953.3 →275.1, respectively). Tolbutamide served as the internal standard (IS), with precursor and product ions at 269.1 →170.1. The parameters were analyzed by nonparametric analysis *via* DAS 3.2.8 software. The regression equation for CHLA, based on the peak area ratio of CHLA to IS against the concentration, was (Eq. (1)):

(1) y=0.0000796x+0.000162(r=0.9968)

indicating a strong correlation. The assay demonstrated a linear response over the concentration range of 10 to 20,000 ng/mL.

### 2.12 Acute toxicity test in mice

Specific-pathogen-free (SPF) C57BL/6 mice were used, balanced for gender, with males and females weighing 18–20 g. The Institutional Committee approved all animal experiment procedures in this study for Animal Care and Biosafety at the Animal Center, Tianjin University of Traditional Chinese Medicine. CHLA was weighed and subsequently dissolved in a 5% DMSO solution, which was then diluted with normal saline (0.9% sodium chloride solution) and mixed to achieve the desired concentration. To evaluate CHLA's acute toxicity, mice were randomly allocated into three groups (*n* = 10): a control group administered normal saline, and two treatment groups administered CHLA solution at 200 and 500 mg/kg, respectively. We monitored all mice that occurred twice daily over 14 days, focusing on toxicological signs, changes in body weight, and behavioral abnormalities. Upon concluding the study, specimens from blood, heart, liver, spleen, lung, and kidney were harvested for analysis.

### 2.13 Statistical analysis

All statistical data analyses were performed in GraphPad Prism 8 software. Statistical significance for each endpoint was determined by unpaired two-tailed Student's *t*-test. Data are expressed as mean ± standard deviation (SD). A significant level of *P* < 0.05 was considered statistically significant for all tests.

## 3 Results

### 3.1 Approbation of intermolecular interaction between CHLA and SARS-CoV-2 M^pro^ determined by FRET, TSA, SPR, and SpS

**Figure 1:**
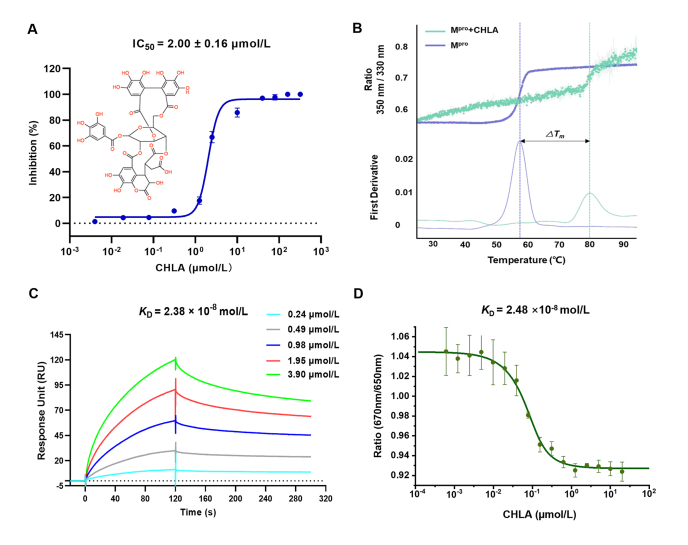
Multi-dimensional strategies for screening SARS-CoV-2 M^pro^ inhibitors. (A) Representative inhibition profile for CHLA against SARS-CoV-2 M^pro^ (200 nmol/L) by FRET assay. (B) The thermal stability of the complex formed by the binding of SARS-CoV-2 M^pro^ (10 μmol/L) with CHLA (500 μmol/L) was determined by a TSA assay. (C) Kinetic profiles of binding affinity between CHLA and SARS-CoV-2 M^pro^ by SPR assay. (D) Spectral shift fluorescence response of CHLA interaction with SARS-CoV-2 M^pro^ (100 nmol/L). Data are shown as mean ± SD (*n* = 3).

The FRET between two molecules is a crucial physical phenomenon with considerable interest for understanding some biological processes at the molecular level, which is sensitive to the distance between the donor and acceptor fluorophore. For the FRET assay, recombinant SARS-CoV-2 M^pro^ with its native N-terminal and C-terminal residues was successfully expressed in *Escherichia coli* and subsequently purified (Supporting Information Fig. S1), which was checked by SDS-PAGE to ensure its purity first. A specific fluorescence-labeled substrate, MCA-AVLQSGFR-Lys(Dnp)-Lys-NH_2_, was synthesized, which can be cleaved by SARS-CoV-2 M^pro^ to quench fluorescence, displaying the enzyme activity according to the dynamic change of fluorescence. The titrated CHLA in the range of 0.004–320 μmol/L can inhibit SARS-CoV-2 M^pro^ activity in a dose-dependent manner by the FRET assay, whose half-maximal inhibitory concentration (IC_50_) value was given at 2.00 ± 0.16 μmol/L (Fig. 1A), displaying the strong inhibition against M^pro^. As a positive control, PF-07321332 was witnessed to perform inhibition activity on M^pro^ with an IC_50_ value at 0.05 μmol/L (Supporting Information Fig. S2) in agreement with the result reported^22^.

TSA is employed to determine the thermal stability of protein, which is improved when the ligand is bound to the protein. As the key indicators, the intensity of fluorescence and Δ*T*_m_ were employed to mirror the mutual interaction between protein and ligand^29^. From Fig. 1B, the melting point of SARS-CoV-2 M^pro^ is greatly elevated from 57.6 to 80.0 °C, and the shift of the emission spectrum happened at the moment of formation of the M^pro^–CHLA complex, suggesting the occurrence of perfect binding between SARS-CoV-2 M^pro^ and CHLA.

SPR and SpS are always performed to determine the intermolecular affinity according to the change in signal. By optimizing sensor chips, association and dissociation times, flow rate, and running buffer^30^, satisfactory SPR experimental conditions were established to qualify the binding affinity between CHLA and SARS-CoV-2 M^pro^. The binding curves were obtained to show the association-dissociation pattern in a dose-dependent style, giving an excellent *K*_D_ value at 2.38 × 10^−8^ mol/L (Fig. 1C). SpS is a novel tool for verifying the interactions by the Monolith X instrument. By recording the fluorescence data, the ratio of signals at 670 and 650 nm was calculated in a dose-dependent manner, yielding *K*_D_ at 2.48 × 10^−8^ mol/L for the SARS-CoV-2 M^pro^–CHLA complex (Fig. 1D). The *K*_D_ values determined by SPR and SpS were almost consistent, displaying a promising antiviral candidate.

### 3.2 Characterization of binding manner between CHLA and SARS-CoV-2 M^pro^*via* saturation transfer difference (STD) NMR and water-ligand observed *via* gradient spectroscopy (WaterLOGSY) spectra

**Figure 2:**
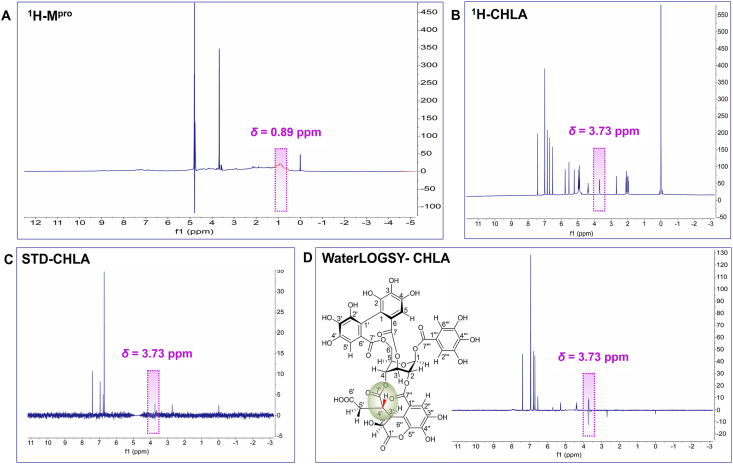
^1^H STD and WaterLOGSY NMR of SARS-CoV-2 M^pro^–CHLA complex. (A) ^1^H NMR spectrum of SARS-CoV-2 M^pro^ (100 nmol/L) (B) ^1^H NMR spectrum of CHLA (10 μmol/L). (C) STD NMR spectrum of SARS-CoV-2 M^pro^ (100 nmol/L)–CHLA (10 μmol/L) complex. (D) WaterLOGSY spectrum of SARS-CoV-2 M^pro^ (1 μmol/L)–CHLA (10 μmol/L) complex.

^1^H STD NMR spectroscopy is performed to define the binding epitope of SARS-CoV-2 M^pro^ inhibitor, which relies on the transfer of saturation from protein to ligand^31^. The signal (*δ*^H^) of M^pro^ was at 0.892 ppm (Fig. 2A), which differs from CHLA ^1^H NMR (Fig. 2B). STD NMR signals (*δ*^H^) were at 2.72, 3.73, 6.71, 6.76, 6.94, and 7.39 ppm (Fig. 2C), which were the potential binding sites, but the exact binding positions of CHLA need to be confirmed.

Similar to STD NMR, WaterLOGSY NMR also relies on magnetization transfer, but the signal from hydrogens of the ligand that interacts with the protein appears to be negative, which is used as an alternative strategy for verification of STD NMR results^32^. As shown in Fig. 2D, the detected negative signals (*δ*^H^) are at 3.73 and 2.68 ppm, which are consistent with the STD NMR data. Compared with the previous study and CHLA ^1^H-NMR^33^, the *δ*^H^ at 2.72 and 2.68 were assigned to the solvent dimethyl sulfoxide (DMSO), but 3.73 ppm is the shared signal in STD and WaterLOGSY NMR, which is located in 4*′*-H of CHLA. Therefore, CHLA is considered to interact with SARS-CoV-2 M^pro^ *via* 4*′*-H.

### 3.3 Evaluation of antiviral activity for CHLA by SARS-CoV-2 strains and variants *in vitro* and *in vivo*

Due to the inherent high mutability in the sequence of SARS-CoV-2 S^pro^, SARS-CoV-2 WT strives for ongoing adaptive evolution and globalization, resulting in accelerating emergence of SARS-CoV-2 variants, such as SARS-CoV-2 Delta, SARS-CoV-2 Omicron BA.1.1, SARS-CoV-2 Omicron BA.2.3, SARS-CoV-2 Omicron BA.4, and SARS-CoV-2 Omicron BA.5. It poses a great challenge to discover the promising candidates.

**Figure 3:**
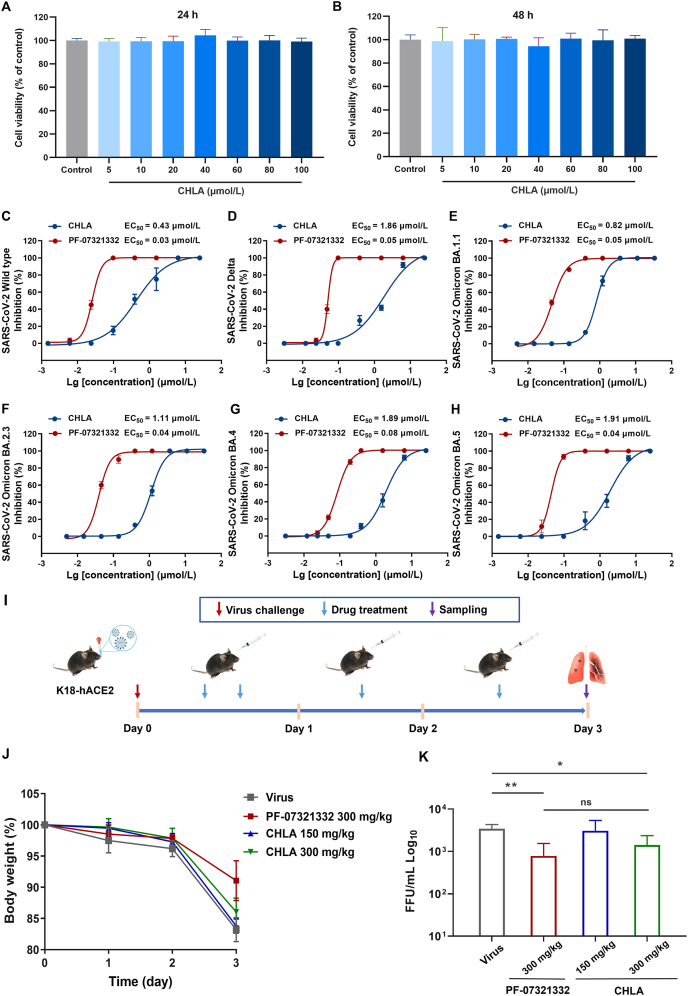
*In vitro* and *in vivo* antiviral efficacy of CHLA as the SARS-CoV-2 M^pro^ inhibitor. (A, B) Cytotoxicity evaluation *in vitro*. (C–H) Antiviral activity of CHLA against SARS-CoV-2 WT strain and variants in cell-based assays. (I) The experimental design in the K18-hACE2 mice. (J) Daily body weight changes of K18-hACE2 mice in different groups (*n* = 5 per group). (K) K18-hACE2 mice lung viral titer determined by standard plaque assay. (*n* = 5 per group). Data are expressed as mean ± SD. ∗*P* < 0.05 and ∗∗*P* < 0.01 *vs* Virus group; ns indicates no significant difference.

Hence, in our study, these strains were focused on the broad-spectrum antiviral drug screening. In cell-based assays, the CCK8 assay was carried out to undertake the cytotoxic evaluation of CHLA in Vero E6 cells for 48 h, which showed that cell viability was not significantly affected compared to the control group, giving CHLA CC_50_ > 100 μmol/L (Fig. 3A and B). Subsequently, in SARS-CoV-2 strain-infected Vero E6 cells, the cytopathic effect (CPE) of the tested compounds was monitored by the Celigo image cytometer to calculate EC_50_ values. As shown in Fig. 3C–H, PF-07321332, a positive control, displays pan-human coronavirus antiviral activity and excellent off-target selectivity *in vitro* and safety profiles *in vivo*^22^, whose EC_50_ was determined at about 50 nmol/L for the tested SARS-CoV-2 strains and variants. CHLA was proved to inhibit SARS-CoV-2 WT with EC_50_ at 0.43 μmol/L (Fig. 3C), SARS-CoV-2 Delta variant at 1.86 μmol/L (Fig. 3D), SARS-CoV-2 Omicron BA.1.1 variant at 0.82 μmol/L (Fig. 3E), SARS-CoV-2 Omicron BA.2.3 variant at 1.11 μmol/L (Fig. 3F), SARS-CoV-2 Omicron BA.4 variant at 1.89 μmol/L (Fig. 3G), and SARS-CoV-2 Omicron BA.5 variant at 1.91 μmol/L (Fig. 3H), which was suggested as a promising candidate for controlling COVID-19.

To evaluate the antiviral efficacy of CHLA *in vivo*, we treated K18-hACE2 mouse models by oral administration with different doses of CHLA (Fig. 3I), which were infected with 1000 FFU of SARS-CoV-2 WT. Notably, the mice treated with CHLA and PF-07321332 at 300 mg/kg exhibited weight recovery (Fig. 3J). For the viral titers study, the CHLA-treated group (300 mg/kg) and PF-07321332-treated group (300 mg/kg) showed a significant decrease in lung live viral titers three days post-infection (*P* < 0.05 *vs* Virus group and *P* < 0.01 *vs* Virus group, respectively) (Fig. 3K). The lower dose of CHLA (150 mg/kg) was demonstrated to be effective, but no significant difference (*P* > 0.05 *vs* Virus group), indicating a dose-dependent response from 150 to 300 mg/kg. Moreover, the efficacy of CHLA at a dose of 300 mg/kg is equivalent to that of PF-07321332 (300 mg/kg), which served as a positive control (*P* > 0.05). Accordingly, these findings suggest that CHLA can effectively reduce live virus titer in the respiratory tract and mitigate SARS-CoV-2 infection *in vivo*.

### 3.4 Structure of SARS-CoV-2 M^pro^ in complex with CHLA

**Figure 4:**
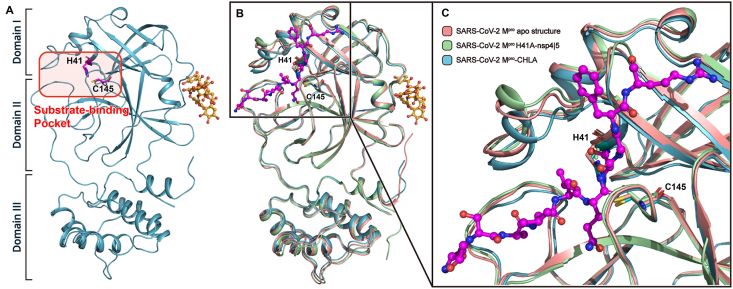
Crystal structure of the SARS-CoV-2 M^pro^–CHLA complex. (A) The overall structure of SARS-CoV-2 M^pro^–CHLA complex. The substrate-binding pocket is labeled with a red rectangle. The catalytic Cys-His dyad is shown as a magenta stick model. The CHLA molecule is shown as a ball-and-stick model with the carbon atoms colored in bright orange and the oxygen atoms in red. (B) The comparison among SARS-CoV-2 M^pro^ in apo form (PDB ID: 6M03, salmon), SARS-CoV-2 M^pro^–CHLA complex (teal), and H41A mutant in nsp4**|**5 peptide binding form (PDB ID: 7DVP, pale green). CHLA and nsp4**|**5 peptide is shown as ball-and-stick models with the carbon atoms colored in bright orange and magenta, respectively. The oxygen atoms and nitrogen atoms are colored red and blue, respectively. (C) The zoomed-in view of the substrate binding pocket.

In order to illustrate the detailed working mechanism of CHLA against SARS-CoV-2 M^pro^, we solved the crystal structure of SARS-CoV-2 M^pro^ in complex with CHLA at 1.4 Å resolution. The complex structure belongs to the *P*2_1_2_1_2 space group, and there is one M^pro^ protomer and one CHLA molecule in an asymmetric unit. There are 304 residues that can be clearly traced according to the electron density map. Consistent with previous studies, the M^pro^ protomer is composed of three domains that are conserved among CoVs. The substrate-binding pocket lies in the cleft between domain I and domain II, featuring a non-canonical Cys-His dyad (C145 and H41) (Fig. 4A). Surprisingly, the ligand is located in a shallow groove at the junction of domain I and domain II that is far away from the substrate-binding pocket. Compared with SARS-CoV-2 M^pro^ H41A-nsp4**|**5 complex structure and apo structure, the substrate binding pocket of SARS-CoV-2 M^pro^–CHLA complex structure is significantly shrunk (Fig. 4B and C). This constricted substrate binding pocket cannot accommodate the nsp4**|**5 peptide and therefore leads to loss of hydrolase activity *in vitro*.

**Figure 5:**
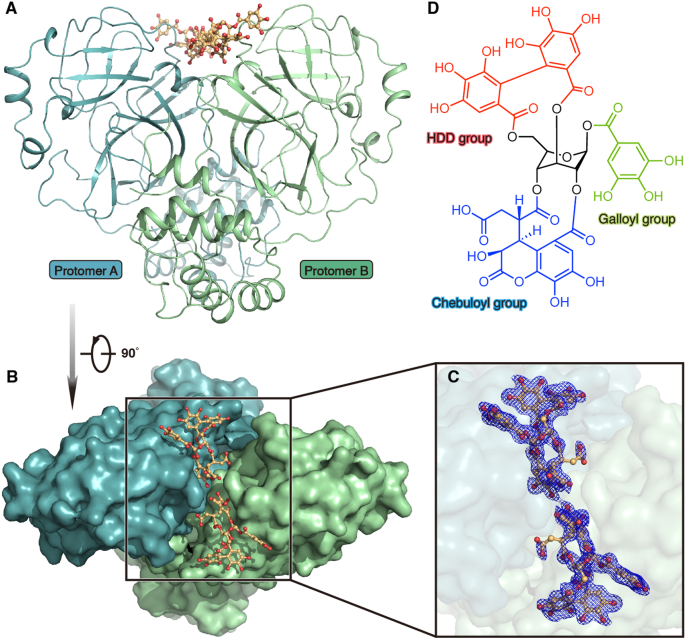
Crystal structure of SARS-CoV-2 M^pro^–CHLA complex in dimer form. (A) The overall structure of the homodimer of SARS-CoV-2 M^pro^–CHLA complex. Protomer A and protomer B are colored in light teal and pale green, respectively. CHLA is shown as a ball-and-stick model with the carbon atoms in bright orange and the oxygen atoms in red. (B) The top view of the homodimer of the SARS-CoV-2 M^pro^–CHLA complex structure. Protomer A and protomer B are shown as surface representations and colored in light teal and pale green, respectively. (C) The zoomed-in view of the CHLA binding site. Two CHLA molecules are shown as ball-and-stick models. The polder electron density map (*F*_o_–*F*_c_) of CHLA is colored in blue mesh and contoured at 2.5*σ*. (D) The chemical structure of CHLA. The central glucose backbone is colored in black. The galloyl group, hexahydroxydiphenoyl (HDD) group, and chebuloyl group are colored in bright green, red, and blue, respectively.

It is well accepted that SARS-CoV-2 M^pro^ releases itself through an autocleavage process and forms an active functional homodimer to accelerate the hydrolysis of polyproteins^14^^,^^15^. In order to further elucidate the inhibitory mechanism of CHLA, we put our vision in a broader perspective to analyze the complex structure. Interestingly, we found two CHLA molecules lie in the cleft formed by two M^pro^ protomers of the functional dimer, enhancing the interaction between them (Fig. 5A–C). There is no significant difference between SARS-CoV-2 M^pro^–CHLA complex dimer and SARS-CoV-2 M^pro^ apo dimer, with a rmsd value of 0.639 Å for all Cα atoms (Supporting Information Fig. S3). However, it should be noted that the structure of the CHLA binding structure has a slight contraction compared with the apo structure (Fig. S3), indicating that the CHLA molecules do tighten the interactions between the two protomers in a functional dimer. In addition, we found that CHLA in the functional homodimer also interacted with the adjacent homodimers. This means that this functional dimer can interact with two adjacent dimers through two CHLA molecules. Therefore, CHLA acts as a lectin-like molecule that may cause SARS-CoV-2 M^pro^ aggregation in this process.

**Figure 6:**
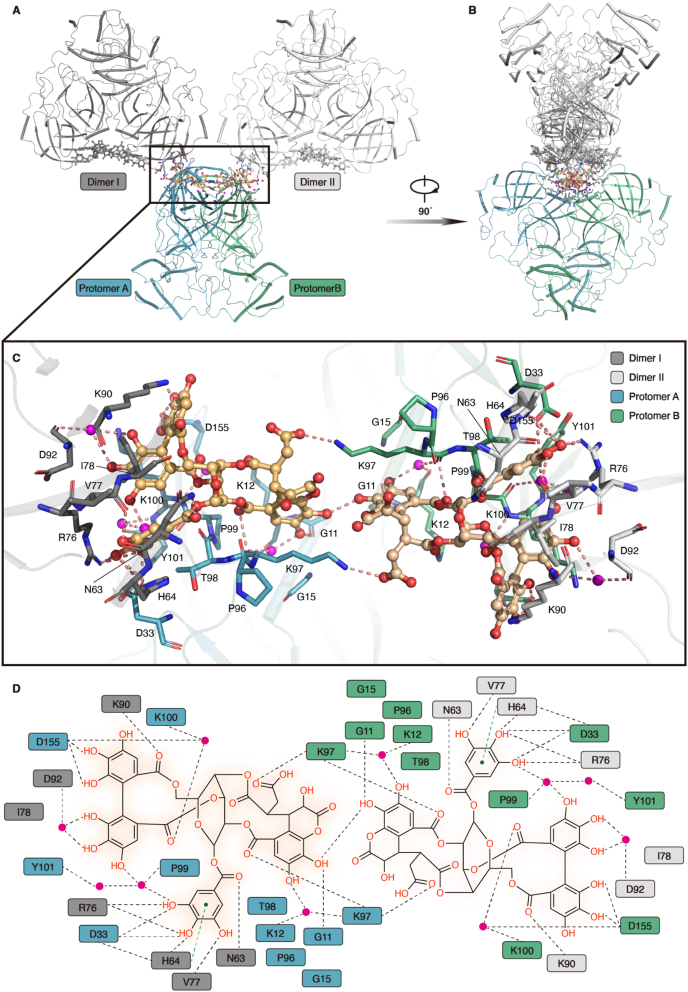
The detailed binding mode of SARS-CoV-2 M^pro^–CHLA complex. (A) The interactions of two protomers in a dimer and the interactions between adjacent dimers are mediated by CHLA. Protomer A and Protomer B are colored in light teal and pale green, respectively. Dimer I and dimer II are colored in dark gray and light gray, respectively. (B) The side view of the interaction model between one M^pro^ dimer and two adjacent dimers. (C) The detailed interaction between SARS-CoV-2 M^pro^ and CHLA. Residues that are involved in substrate binding are shown as colored sticks. Ordered water molecules are represented in a magenta sphere. (D) The hydrogen bond networks between SARS-CoV-2 M^pro^ and CHLA. Residues that form hydrogen bonds with SARS-CoV-2 M^pro^ are represented in colored squares. Ordered water molecules are represented in the magenta cycle.

As a natural ellagitannin from myrobalans, in addition to the central glucose backbone, the CHLA molecule can be divided into three parts: the galloyl group, the hexahydroxydiphenoyl (HDD) group, and the chebuloyl group (Fig. 5D). The chebuloyl group is mainly related to the dimerization of protomer A and protomer B, while the other two parts are involved in the interactions between dimers (Fig. 6A and B). The connection between M^pro^ protomer and CHLA molecule, which are both in the same asymmetric unit, is stabilized by numerous hydrogen bond interactions (Fig. 6C and D). In detail, the galloyl group forms two hydrogen bonds with the side chain of D33; the chebuloyl group forms one hydrogen bond with the backbone carboxyl oxygen of residues G11 and K97, respectively; and the HDD group also forms two hydrogen bonds with the side chain of D155. Additionally, the galloyl group interacts with the backbone carboxyl oxygen of P99, the main chain NH group of Y101, and the HDD group through two water molecules; the chebuloyl group interacts with a water molecule bridging the backbone carboxyl oxygen of residues K12 and K97. Meanwhile, the HDD group also interacts with the main chain NH group of K100 and the backbone carboxyl group of D155 through a hydrogen bond formed with another ordered water.

There are three additional hydrogen bonds formed, enhancing the interactions between two M^pro^ protomers (Fig. 6C and D). The first hydrogen bond is formed between the chebuloyl group of CHLA from each protomer within the same functional dimer. Two other hydrogen bonds are formed between the chebuloyl group of CHLA from one protomer and K97 from another protomer. The chebuloyl group from protomer A interacts with the side chain of K97 from protomer B, and vice versa for the chebuloyl group from protomer B, which interacts with the side chain of K97 from protomer A.

CHLA molecules from different protomers within the functional dimer interact with their corresponding nearest dimers using the same binding mode (Fig. 6C and D). For example, protomer A from one dimer interacts with domain I of protomer A from another dimer, mediated by CHLA. Specifically, the interactions between these two protomers are primarily mediated by the galloyl group and the HDD group of CHLA. The galloyl group interacts with the N*δ*2 atom of N63, the N*η*1 atom of R76, and the backbone carboxyl oxygen of V77 from dimer I, forming four hydrogen bonds. Additionally, the phenyl ring of the galloyl group interacts with the imidazole ring of H64, forming a strong *π*–*π* interaction. In contrast, the HDD group forms one hydrogen bond directly with the side chain of K90 and two hydrogen bonds with the backbone carboxyl oxygen of D92 through an ordered water molecule.

Furthermore, the dynamic light scattering (DLS) analysis was employed to investigate the state of SARS-CoV-2 M^pro^ in the presence of CHLA, which revealed a significant increase in particle size upon the addition of CHLA to M^pro^. Specifically, the cumulant radius of M^pro^ alone is 3.8 nm, while the cumulant radius of M^pro^–CHLA complex is 25.85 nm, indicating that CHLA might promote M^pro^ aggregation. Additionally, the polydispersity index (PDI) for M^pro^ was initially recorded at 0.48, which increased to 1.14 upon CHLA addition, suggesting a more heterogeneous aggregation pattern (Supporting Information Fig. S4).

### 3.5 Druggability evaluation of CHLA

Pharmacokinetics (PK) and toxicology profiles are the key indicators for druggability evaluation, which have a great impact on the development of new drugs, especially oral drugs. The satisfactory PK and safety prove that the tested compound has potential for oral administration, paving the way for new drug development.

**Figure 7:**
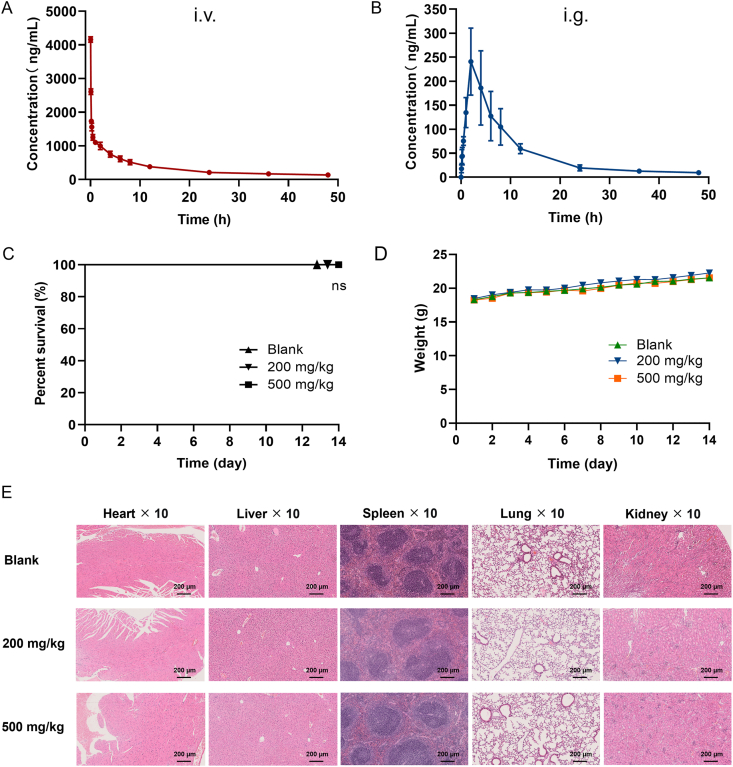
Evaluation of CHLA's Druggability. (A) Time–concentration profiles of CHLA in rats (*n =* 5, i.v.). (B) Pharmacokinetic profiles of CHLA in rats (*n =* 5, i.g.). (C) Survival curves for mice receiving CHLA (*n =* 10, i.g.). (D) Body weight trajectories over 14 days for C57BL/6 mice across various treatment groups (*n =* 10, i.g.). (E) Histological analysis for the key tissues at different dosages (200 and 500 mg/kg, administered *via* i.g.).

By liquid chromatography coupled to tandem mass spectrometry (LC–MS/MS), a study on the oral PK profile was performed after the systematic methodological validation. With a single dose at 10 mg/kg to SD rats (i.g.), CHLA was witnessed to give a preferred oral PK profiling with 15.2 h for half-life (*t*_1/2_), 240.7 μg/L for maximal concentration (*C*_max_), 2323.5 μg h/L for the area under the curve (AUC_*0-t*_), and 0.001 L/h/kg for the clearance (CL) (Fig. 7A). Compared with intravenous injection administration (i.v.) at 5 mg/kg to SD rats (Fig. 7B), the oral bioavailability (*F*%) was given at 7.1%, which suggested that CHLA had a prospect for oral antiviral drugs development (Supporting Information Table S2).

Regarding the cell membrane permeability of CHLA, we employed permeability assays with Caco-2 cells to conduct transport studies in both apical-to-basolateral (A→B) and basolateral-to-apical (B→A) directions, unveiling the entry mechanism of CHLA into cells and evaluating its intracellular bioavailability. Atenolol and minoxidil served as positive controls to characterize low and high absorptive permeabilities with the apparent permeability coefficient (*P*_app_), respectively. The *P*_app(A–B)_ of atenolol was recorded at 0.28 × 10^−6^ cm/s, indicating low permeability, whereas minoxidil exhibited a higher permeability with *P*_app(A–B)_ recorded at 5.94 × 10^−6^ cm/s. At a concentration of 5 μmol/L CHLA, the *P*_app(A–B)_ was 1.20 × 10^−6^ cm/s, and *P*_app(B–A)_ was 0.39 × 10^−6^ cm/s, and the efflux ratio was 0.33, suggesting a moderate membrane permeability (Supporting Information Table S3).

An acute toxicity test for oral administration of CHLA was undertaken on C57BL/6 mice at doses of 200 mg/kg and 500 mg/kg (Fig. 7C). CHLA was proven to be safe. The body weight of mice steadily increased with no significant difference observed compared with the control group (Fig. 7D). No obvious histological changes were observed in the important tissues, such as the lung, heart, spleen, liver, and kidney (Fig. 7E).

## 4 Discussion

Given the central importance of SARS-CoV-2 M^pro^ in viral replication, its potential for ensuring mechanistic safety, and the expected lack of spike protein variant resistance challenges, M^pro^ emerges as an exceptionally promising target for oral antiviral therapy to treat COVID-19. There exist two primary classes of inhibitors aiming at cysteine proteases: covalent inhibitors and non-covalent inhibitors. The development of covalent inhibitors typically presents a challenge due to their potential toxicity, lack of specificity, and consequences of irreversible modification of non-target cysteine residues. Nevertheless, advanced strategies, such as rational design, have been employed to achieve selective covalent inhibition of M^pro^^15^^,^^34^. Conversely, non-covalent inhibitors reversibly bind to their target proteins with high specificity, exhibiting a superior ability to penetrate membranes and bind to the target protein with high affinity. Moreover, owing to their absence of reactive functional groups or ‘warheads’, non-covalent inhibitors facilitate the reversible interactions with proteins and nucleic acids, highlighting their promising drug-like properties^35^, ^36^, ^37^, ^38^.

In the quest to develop SARS-CoV-2 inhibitors, natural products have emerged as a significant reservoir of therapeutic potential, exhibiting a variety of mechanisms of action. Focusing on SARS-CoV-2 M^pro^, numerous active compounds bind to the classical substrate pocket as the non-canonical C145-H41 dyad, such as shikonin^19^. Previous studies have identified CHLA as a potential SARS-CoV-2 M^pro^ inhibitor, and the docking analyses revealed its affinity for an allosteric site, which is a highly reactive pocket within the enzyme's dimerization interface at domain Ⅲ^39^. However, a notable gap in understanding has been the absence of crystal complex structures elucidating the binding mode.

Our research advances the field by presenting the crystal complex of SARS-CoV-2 M^pro^-CHLA, which was resolved at an impressive 1.4 Å resolution. Contrary to expectations, CHLA does not accommodate the enzyme's substrate binding site or previously reported allosteric sites, but binds within a shallow groove located at the interface between domain I and domain II. This binding site is distinct from the active site targeted by inhibitors like Nirmatrelvir. As reported, the E166V mutation of M^pro^ exhibited drug resistance to Nirmatrelvir, significantly reducing the binding affinity and consequently diminishing its inhibitory efficacy by over 200-fold due to disrupted interactions^40^. Notably, CHLA's unique binding site, away from the classical active site, is promising to avoid drug resistance due to the E166V mutation in SARS-CoV-2, highlighting the potential strategic benefit.

In the docking study, when CHLA binds to SARS-CoV-2 M^pro^ at the novel site, there is no interaction between the nsp4|5 peptide substrate (TSAVLQ|SGFR) and the classical binding pocket due to the shrinkage of the binding pocket caused by CHLA. This newly identified shallow groove signifies an unconventional allosteric site, possibly contributing to CHLA's antiviral efficacy. Such a mechanism of action diverging from classical active site inhibition potentially paves the way for alternative therapeutic strategies.

**Figure 8:**
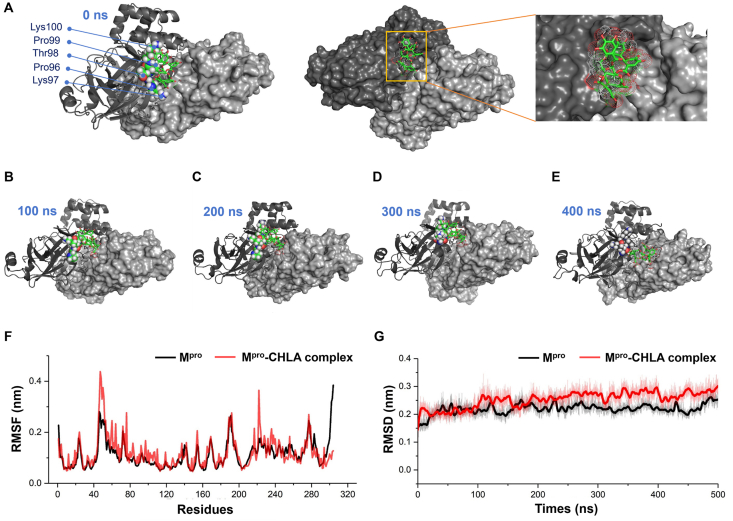
Illustration of CHLA's conformations inside the allosteric binding site of SARS-CoV-2 M^pro^. (A) CHLA's conformation in the molecular dynamics simulation (MD) study at 0 ns. The main residues interacting with CHLA are shown as blue, CHLA colored green, and SARS-CoV-2 M^pro^ dimer colored gray. (B–E) CHLA's conformation in the MD trajectory at 100 ns (B), 200 ns (C), 300 ns (D), and 400 ns (E). The results of RMSD (F) and RMSF (G) between SARS-CoV-2 M^pro^ and CHLA.

In the preliminary phase of screening for active compounds, molecular docking stands out as an efficient tool for high-throughput virtual screening. With the advent of AI technology, the efficiency and accuracy of virtual screening have improved greatly. Nonetheless, on account of the inherent model limitations, the results of the simulation may not accurately reflect the actual physiological state. Active SARS-CoV-2 M^pro^ is characterized as a homodimer including two protomers. Given the structural symmetry of protein units, a single protomer is utilized as a receptor for ligand screening, which diverges from the M^pro^ active conformation. The shallow groove identified in this study is formed by two protomers. In the molecular dynamics study, this newly discovered groove is designated as the binding site, and we endeavor to ascertain the stable conformation of the SARS-CoV-2 M^pro^–CHLA complex within a protomer. Unfortunately, CHLA exhibits an unstable binding pattern in a single protomer. However, CHLA was demonstrated to stably bind with an M^pro^ dimer over a span of 500 ns (Fig. 8 and Supporting Information Fig. S5), proving the reliability of configuring the protein in its active state for docking simulations.

PF-07321332 has demonstrated promising outcomes in preclinical trials and has progressed to clinical phases, underscoring its capability to significantly diminish viral replication^41^^,^^42^. By specifically targeting SARS-CoV-2 M^pro^, PF-07321332 disrupts the viral replication cycle, effectively curbing viral replication within the host^43^. Therefore, in this study, we introduce PF-07321332 as the positive control. Notably, the antiviral efficacy of CHLA at a dosage of 300 mg/kg was observed to be comparable to that of PF-07321332 administered at an equivalent dose (*P* > 0.05). In addition, CHLA displayed superior solubility, forming a clear solution at the effective dose (300 mg/kg), in contrast to PF-07321332, which exhibited poor solubility and remained in suspension. This attribute enhances CHLA's drug-like properties, suggesting its viability as a potent anti-SARS-CoV-2 inhibitor.

CHLA was predominantly extracted from *Terminalia Chebula*, which is acknowledged as an economical and natural antiviral agent. It exhibits efficacy against herpes simplex virus types 1 (HSV-1)^44^ and 2 (HSV-2)^45^, as well as various strains of influenza A virus^46^. Due to its effect on human immunodeficiency virus (HIV)^47^ and hepatitis C virus (HCV)^48^ in clinical practice, CHLA is advocated as an alternative therapy for COVID-19^39^^,^^49^. Encouragingly, our study reveals that CHLA demonstrates a promising antiviral activity against both SARS-CoV-2 WT strain and several of its variants. Despite these variants exhibiting variations in S^pro^ sequences, minimal alterations are observed in M^pro^, underlining the potential for CHLA's broad-spectrum inhibitor. The detailed investigation into its structure-activity relationships, coupled with its unique mechanism of action on M^pro^, supports its promising antiviral performance and favorable safety profile. Consequently, CHLA is proposed as a promising candidate for antiviral intervention, underscoring its clinical potential in COVID-19 management. Also, the screening model was successfully proven for targeting SARS-CoV-2 M^pro^ for the discovery of a broad-spectrum COVID-19 inhibitor by intensive multi-tiered validation.

## 5 Conclusions

Overall, the present study provides a practical approach for identifying bioactive compounds targeting specific biomolecules. The discovery of a novel allosteric site on SARS-CoV-2 M^pro^ expands the horizon for inhibitor screening, while the identification of CHLA as the allosteric inhibitor offers valuable insights into the structural modification of potential therapeutic agents.

## Data availability

Coordinates and structure factors have been deposited in Protein Data Bank (PDB) with accession number 8ZBP. X-ray data collection and refinement statistics are given in Table S1. All data are available in the main text or the Supporting Information.

## Author contributions

Boli Zhang, Zihe Rao, Junhua Zhang and Yuefei Wang: Conceptualization, Supervision. Yao Zhao, Junhua Zhang and Yuefei Wang: Supervision, Formal analysis, Funding acquisition, Writing- Reviewing and Editing. Min Zhang and Changjian Wang: Methodology, Software, Data curation, Writing-Original draft preparation. Lu Feng: Visualization, Investigation. Qi Yang and Yipeng Cao: Software, Validation.

## Conflicts of interest

The authors declare no conflicts of interest.

## Appendix A Supporting information

The following is the Supporting Information to this article.
