# Supplementary material for: Targeting SARS-CoV-2 main protease for the discovery of a broad-spectrum COVID-19 inhibitor by intensive multi-tiered validation
Source: Acta Pharm Sin B. 2025 Sep 22;15(11):5789–802. doi: 10.1016/j.apsb.2025.09.033 (PMC12648101; doi:10.1016/j.apsb.2025.09.033)
Supplement: Multimedia component 1 [file mmc1.pdf]

Supporting Information for

Original article

Targeting SARS-CoV-2 main protease for the discovery of a broad-spectrum COVID-19 inhibitor by intensive multi-tiered validation

Min Zhang[a,b,†], Changjian Wang[a,†], Lu Feng[c,†], Qi Yang[d], Yipeng Cao[e], Yao Zhao[f,g,*], Junhua Zhang[a,b,*], Yuefei Wang[a,b,*], Zihe Rao[c,g,h], Boli Zhang[a,b]

[a]*State Key Laboratory of Chinese Medicine Modernization, Tianjin University of Traditional Chinese Medicine, Tianjin 301617, China*

[b]*Haihe Laboratory of Modern Chinese Medicine, Tianjin 301617, China*

[c]*State Key Laboratory of Medicinal Chemical Biology, Frontiers Science Center for Cell Response, College of Life Sciences, College of Pharmacy, Nankai University, Tianjin 300071, China*

[d]*Guangzhou Laboratory, Guangzhou 510005, China*

[e]*Key Laboratory of Cancer Prevention and Therapy, Tianjin's Clinical Research Center for Cancer, National Clinical Research Center for Cancer, Tianjin Medical University Cancer Institute and Hospital, Tianjin 300060, China*

[f]*National Clinical Research Center for Infectious Disease, Shenzhen Third People's Hospital, Shenzhen 518112, China*

[g]*Shanhai Institute for Advanced Immunochemical Studies and School of Life Science and Technology, ShanghaiTech University, Shanghai 200031, China*

[h]*Laboratory of Structural Biology, School of Life Sciences and School of Medicine, Tsinghua University, Beijing 100084, China*

Received 1 March 2025; received in revised form 6 May 2025; accepted 19 June 2025

*Corresponding authors.

E-mail addresses: zhaoyao@shanghaitech.edu.cn (Yao Zhao), zjhtcm@foxmail.com (Junhua Zhang), wangyf0622@tjutcm.edu.cn (Yuefei Wang).

[†]These authors made equal contributions to this work.

1. Supporting methods

1.1. System construction and simulation

The structure of SARS-CoV-2 M^{pro} dimer is obtained from the PDB database (ID: 6M03). The crystal complex structure of M^{pro}–CHLA is based on our results (PDB ID: 8ZBP). We constructed two simulation systems using the CHARMM-GUI server,

representing the wild-type M^{pro} and CHLA-M^{pro} dimer structures. These systems include M^{pro} dimer, water molecules, ions, and CHLA. The simulation system dimensions are 12 nm×12 nm×12 nm, with a total atom count of ~100,000.

1.2. MD simulation

Similar to the simulation parameters used in previous studies[1,2]. All-atom force fields employed CHARMM36m[3]. A physiological environment was simulated by adding 0.15 mol/L NaCl to the simulation systems. During the simulation stages, an initial energy minimization of 10–20 k steps was performed using the steepest descent algorithm. Subsequently, six equilibration cycles of 10 ns each were conducted, gradually releasing positional constraints on all molecules. The pre-equilibrated systems were then optimized, followed by a 50 ns NPT simulation. Finally, molecular dynamics simulations of the M^{pro} systems in both setups were carried out for 500 ns. The molecular dynamics (MD) time step was set to 2 fs. Electrostatic interactions were described using the Particle Mesh Ewald (PME) algorithm with a cutoff radius of 1.2 nm. Chemical bonds were constrained using the LINear Constraint Solver (LINCS) algorithm. The pressure in the *x*, *y*, and *z* directions was maintained at 1 bar. The temperature was kept at 310 K using the V-rescale thermostat.

1.3. Data analysis

We employed the Molecular Mechanics Poisson-Boltzmann Surface Area (MMPBSA) method to calculate the binding energy between individual proteins in the M^{pro} dimer. For both systems, stable snapshots were extracted from the simulation trajectories for binding free energy calculations, covering a total of 300 ns. A total of 100 snapshots were analyzed at intervals of 3 ns[4]. In this analysis, one M^{pro} protein was designated as the ligand, and the other as the receptor, with their binding free energy represented by Eqs. (1–4):

$$\Delta G_{\text{bind,aq}} = \Delta H - T\Delta S = \Delta G_{\text{complex}} - [\,\Delta G_{\text{protein}} + \Delta G_{\text{ligand}}\,] \quad (1)$$

$$\Delta G_{\text{bind,aq}} = \Delta E_{\text{MM}} + \Delta G_{\text{bind,solv}} - T\Delta S \quad (2)$$

$$\Delta E_{\text{MM}} = \Delta E_{\text{Covalent}} + \Delta \text{E}_{\text{Elect}} + \Delta E_{\text{VDW}} \quad (3)$$

$$\Delta G_{\text{bind,solv}} = \Delta G_{\text{polar}} + \Delta G_{\text{non-polar}} \quad (4)$$

The $\Delta G_{\text{protein}}$ and ΔG_{ligand} are defined as the two monomers of the M^{pro} protein dimer. The binding energy for each residue is decomposed to calculate the contribution of each residue to the binding of the M^{pro} protein dimer.

1.4. Permeability of CHLA in Caco-2 cells

Caco-2 cells were seeded on 0.4 μm pore polycarbonate (PC) membranes in 96-well Corning Transwell insert plates at a density of 2.4 × 10^5 cells/cm^2. Hank's Balanced Salt Solution (HBSS) supplemented with 10.0 mmol/L HEPES (pH 7.40) was used as the transport buffer. CHLA was subjected to bidirectional transport assays in triplicate at a concentration of 5.00 μmol/L. The plates were incubated in a humidified CO_2 incubator (5% CO_2, 37.0 °C) for 2 h under static conditions. Following sample collection, all samples were immediately combined with acetonitrile containing an internal standard and centrifuged at 3220 × *g* for 30 min. The concentrations of CHLA in the initial solution, donor compartment, and receiver compartment were quantified using liquid chromatography-tandem mass spectrometry (LC–MS/MS) based on the peak area ratio of the analyte to the internal standard. After the transport assay, monolayer integrity was assessed using the Lucifer Yellow rejection assay.

1.5. Dynamic light scattering assay

To investigate the aggregation state of M^{pro} in the presence of CHLA, dynamic light scattering (DLS) measurements were conducted using a NanoTemper Technologies instrument. M^{pro} was prepared in a buffer containing 20 mmol/L Tris-HCl (pH 8.0) and 150 mmol/L NaCl. CHLA was initially dissolved in DMSO and subsequently mixed with the M^{pro} solution to achieve final concentrations of 10 μmol/L for M^{pro} and 500 μmol/L for CHLA. The mixture was then incubated for 15 min at 25 °C before measurement. DLS was utilized to assess changes in particle size and distribution, indicated by the cumulant radius and polydispersity index (PDI).

2. Supporting figures

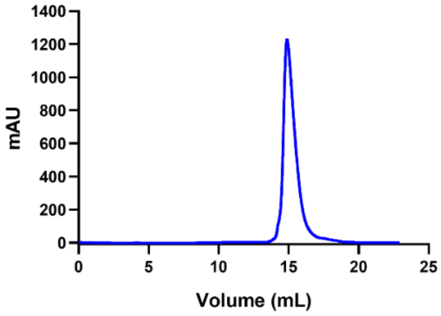

Figure S1 The purification of SARS-CoV-2 M^{pro}.

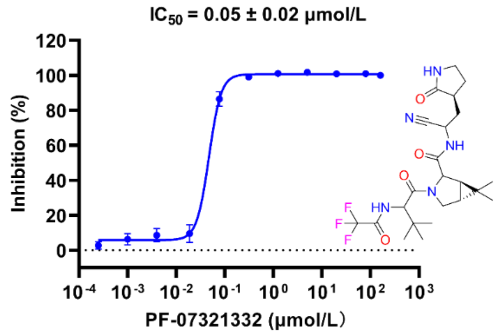

Figure S2 IC_{50} values of PF-07321332 (n = 3).

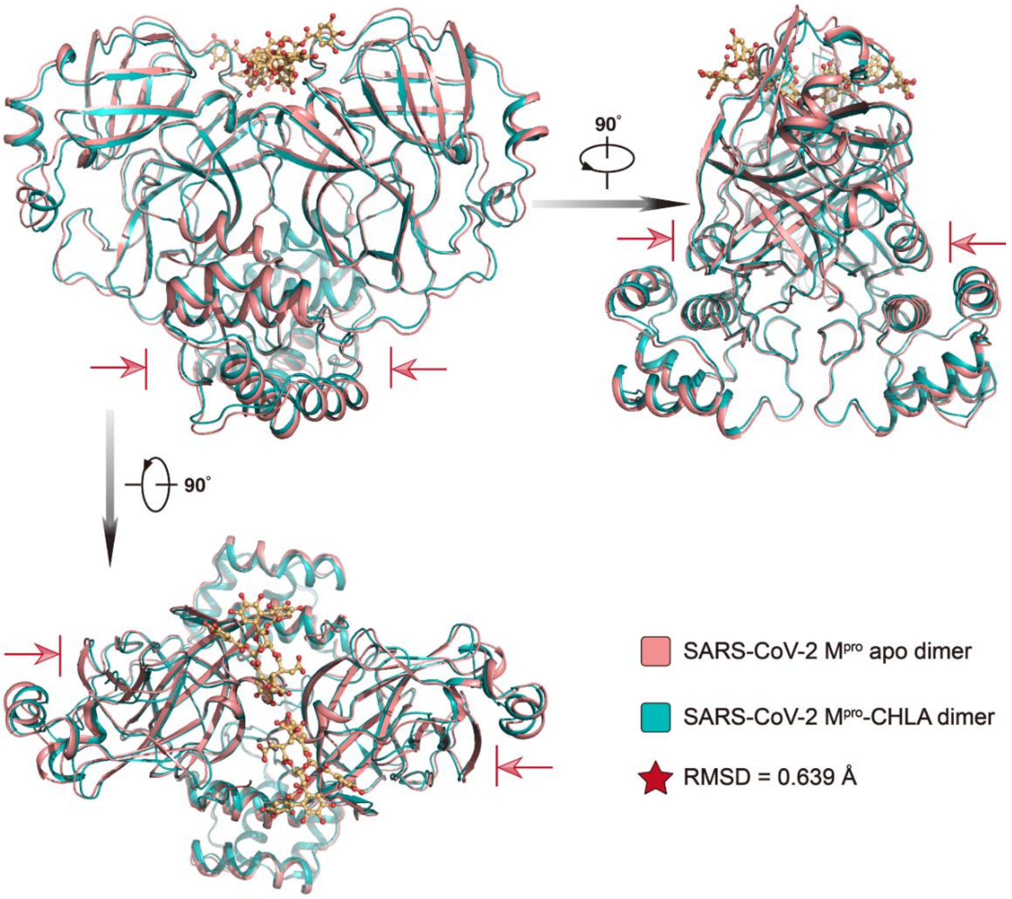

Figure S3 The comparison of SARS-CoV-2 M^{pro} apo structure and SARS-CoV-2 M^{pro}–CHLA complex structure in dimer form. SARS-CoV-2 M^{pro} apo dimer and SARS-CoV-2 M^{pro}–CHLA dimer are colored in salmon and light teal ribbon, respectively. The CHLA molecule is shown as a ball-and-stick model with the carbon atoms colored in bright orange, and oxygen atoms in red.

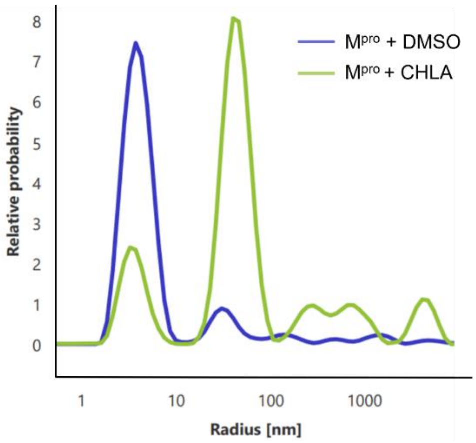

Figure S4 Dynamic light scattering analysis for M^{pro} aggregation dynamics.

3. Supporting results for molecular dynamics simulations

To evaluate and compare the structural stability of M^{pro} dimer in two different states, all-atom molecular dynamics simulations were conducted for each system over a duration of 500 ns. The stability of the models was assessed by analyzing the Root Mean Square Deviation (RMSD). The RMSD of M^{pro} dimer in both systems converges during the simulation process. In the absence of a ligand, the RMSD for the M^{pro} dimer stabilizes around 0.2 nm, with a fluctuation range of only ±0.02 nm, indicating a high level of stability. For M^{pro} dimer bound to the CHLA ligand, the RMSD remains stable at around 0.25 nm, with a fluctuation range of ±0.04 nm. While the RMSD values suggest stability in both systems, M^{pro} dimer bound to CHLA exhibits a higher RMSD and relatively larger fluctuations compared to M^{pro} dimer in its normal state. This suggests a decrease in overall stability for M^{pro} dimer after binding the small molecule.

To characterize the dynamic behavior of each amino acid in the M^{pro} in both simulated systems, we analyzed the Root Mean Square Fluctuation (RMSF) for all systems. Consistent with the RMSD results, the RMSF values for amino acids in M^{pro} dimer without a ligand are smaller in the normal state. When CHLA binding with M^{pro} dimer, the RMSF at the binding site of the dimer increases from ~0.05 to 0.1 nm, suggesting that CHLA has a certain impact on the stability of M^{pro} dimer. These results are consistent with the RMSD.

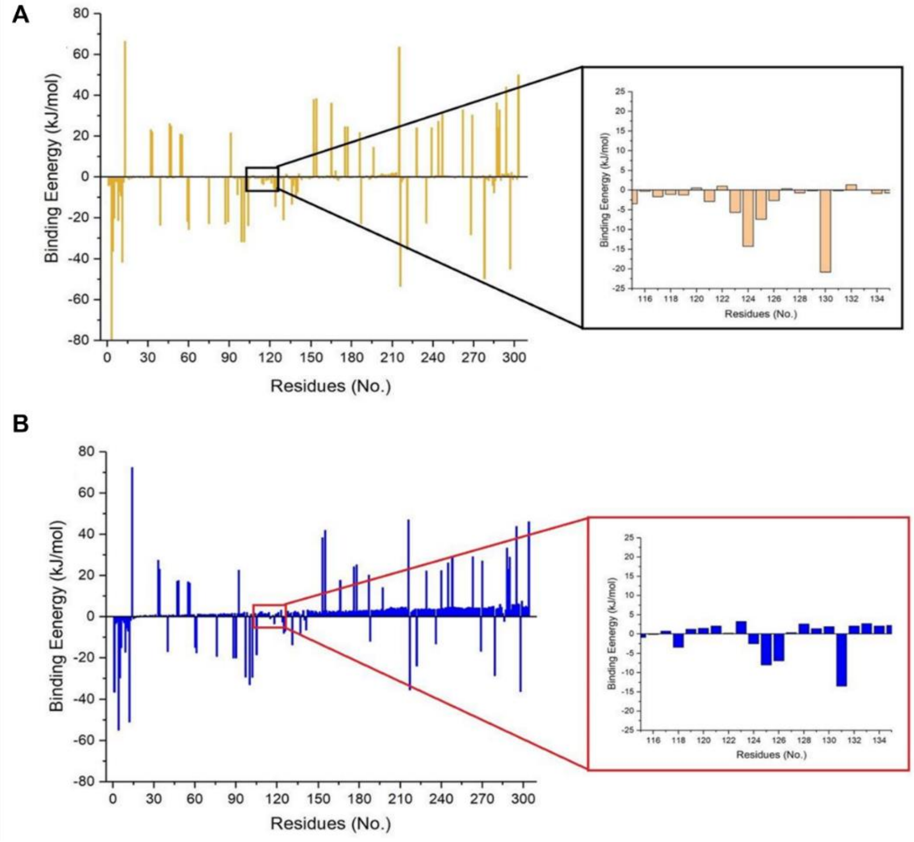

Figure S5 Binding energy (MMPBSA) of SARS-CoV-2 M^{pro} dimers. (A) The binding energy plot for each amino acid of the M^{pro} with no ligand. (B) Binding energy of amino acids of M^{pro} dimer with CHLA molecules. Black and red boxes indicate the binding free energy of the interacting residues (AA: 120–130) between dimers.

We employed the Molecular Mechanics Poisson-Boltzmann Surface Area (MMPBSA) method to investigate the binding energetics of SARS-CoV-2 M^{pro} dimer. The binding energy was decomposed into various components, including van der Waals, electrostatic, polar solvation, and solvent-accessible surface area (SASA). By analyzing 500 ns of simulation trajectories, we calculated the binding energy for all residues (AA: 1–304) in M^{pro}. The results of the MMPBSA analysis indicate a notable alteration in the free energy of M^{pro} dimer when unbound and bound with CHLA. Analyzing the residues involved in their interactions, in the normal state, the binding free energy for residues 120–130 of M^{pro} dimer is negative. The maximum values are observed for S123, G124, V125, and Y126, with values of approximately –5 kJ/mol, –15 kJ/mol, –7 kJ/mol, and –3 kJ/mol, respectively. Upon binding to CHLA, the binding free energy for these four amino acids increases significantly, with values of –2 kJ/mol, –3 kJ/mol,

–8 kJ/mol, and –6 kJ/mol, respectively. This suggests a notable decrease in the binding affinity at the interaction sites between these two systems.

A comprehensive analysis of the RMSD, RMSF, and MMPBSA calculation results suggests that CHLA has a notable impact on reducing the binding affinity of M^{pro} dimer, potentially leading to a decrease in the overall structural stability of the dimer.

4. Supporting tables

Table S1 Data collection and refinement statistics.

**Data Collection**	**M^{pro}-CHLA**
	PDB code: 8ZBP
Space group	$P2_12_12$
Wavelength (Å)	0.9792
Cell dimensions	
a, b, c (Å)	43.34, 61.48, 104.84
α, β, γ (°)	90, 90, 90
Resolution (Å)	39.89–1.41 (1.46–1.41) [a]
No. of unique reflections	54869 (5396)
Completeness (%)	99.93 (99.98)
R_{merge}	0.081 (2.197)
Mean $I/\sigma I$	18.09 (2.23)
*CC*1/2	0.999 (0.512)
Redundancy	12.7 (11.2)
Wilson B factors (Å^2)	17.09
**Refinement**	
Resolution (Å)	39.89–1.41
No. of reflections used	54849 (5396)
R_{work}/R_{free} (%)	19.01/20.71
No. atoms	
Protein	2391
Ligand/ion	72
Water	315
B-factors (Å^2)	
Protein	28.22
Ligand/ion	42.25
Water	37.82
R.m.s. deviations	
Bond lengths (Å)	0.016
Bond angles (°)	1.89
Ramachandran plot (%)	
Favored (%)	98.01
Allowed (%)	1.99
Outliers (%)	0

[a]Values in parentheses are for highest-resolution shell.

Table S2 Preliminary pharmacokinetic (PK) evaluation of CHLA in rats.

Compd.	Admin	AUC_{0-t} (μg·h /L)	C_{max} (μg/L)	$t_{1/2}$ (h)	T_{max} (h)	CLz (L/h/kg)	*F* (%)
CHLA	i.v. (5 mg/kg)	16342.3 ± 1977.1	4160.0 ± 79.4	39.6±13.5	0.033	0.008 ± 0.003	–
	i.g. (10 mg/kg)	2323.5 ± 663.7	240.7 ± 69.83	15.2 ± 7.0	2	0.001	7.1

Table S3 Permeability results of test compounds in Caco-2 cell line.

Compd.	Concentration (μmol/L)	$P_{\text{app (A–B)}}$ (10^{-6}, cm/s)	$P_{\text{app (B–A)}}$ (10^{-6}, cm/s)	Efflux ratio
Atenolol	5	0.28	0.37	1.30
Digoxin	5	0.68	20.22	29.64
Minoxidil	5	5.94	5.71	0.96
CHLA	5	1.20	0.39	0.33

References

1 Cao Y, Wei H, Jiang S, Lu T, Nie P, Yang C, et al. Effect of AQP4 and its palmitoylation on the permeability of exogenous reactive oxygen species: Insights from computational study. *Int J Biol Macromol* 2023: **253**: 127568.

2 Cao Y, Yang R, Wang W, Jiang S, Yang C, Liu N, et al. Probing the formation, structure and free energy relationships of M protein dimers of SARS-CoV-2. *Comput Struct Biotechnol J* 2022; **20**: 573-82.

3 Huang J, Rauscher S, Nawrocki G, Ran T, Feig M, de Groot BL, et al. CHARMM36m: an improved force field for folded and intrinsically disordered proteins. *Nat Methods* 2017; **14**: 71-3.

4 Valdés-Tresanco MS, Valdés-Tresanco ME, Valiente PA, Moreno E. gmx_MMPBSA: a new tool to perform end-state free energy calculations with GROMACS. *J Chem Theory Comput* 2021; **17**: 6281-91.
